# Supplementary material for: Identification of photoperiod-induced specific miRNAs in the adrenal glands of Sunite sheep (Ovis aries)
Source: Front Vet Sci. 2022 Jul 22;9:888207. doi: 10.3389/fvets.2022.888207 (PMC9354845; doi:10.3389/fvets.2022.888207)
Supplement: Supplementary file 1 [file Data_Sheet_1.ZIP › Supplementary file/miRDeep2 file.zip!/miRDeep2 file/Know_Structure/LP42A2/oar-mir-377.pdf]

miRBase precursor : oar-mir-377
Total read count : 22
oar-miR-377-5p read count : 11
oar-miR-377-3p read count : 0
remaining reads : 11

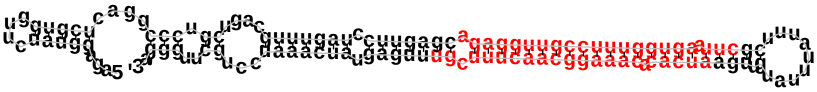

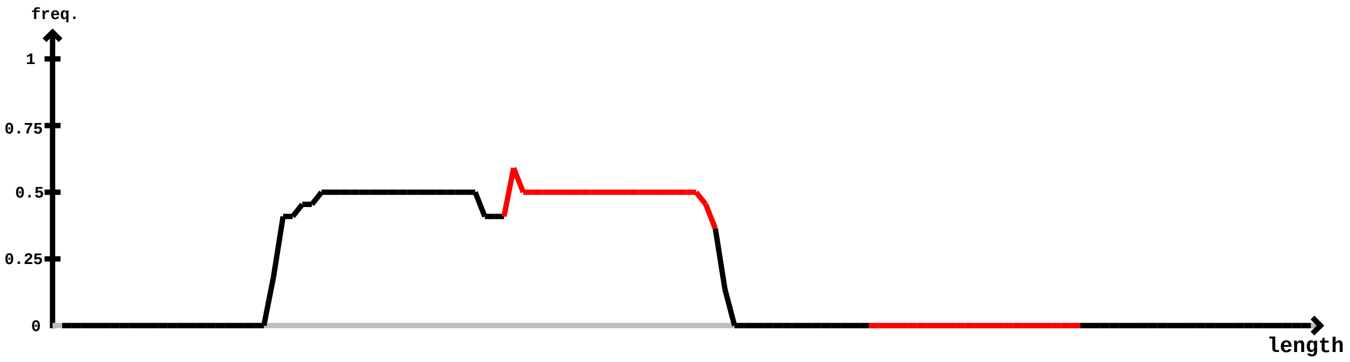


```
                                                            oar-miR-377-3p
                                             oar-miR-377-5p
5'- agagguaucuuggugcucaggcccugcugacguuugauccuugagcagagguugccuuuggugaauucgcuuuauuuauguugaaucacacaaaggcaacuuucguuugaguaucaaauccugcuuggga  -3'  exp
    ...(((((....)))))....(((.((....(((((((.(((((((.(((((((((((((((((.(((((.........).)))))))).))))))))))))).))))))).)))))))...))..))).  reads  mm
    .....................cccugcugacguuugauccuug.......................................................................................  1  0
    .....................cccugcugacguuugauccuugagc....................................................................................  3  0
    ......................ccugcugacguuugauccuug.......................................................................................  1  0
    ......................ccugcugacguuugauccuugagc....................................................................................  2  0
    ......................ccugcugacguuugauccuugagca...................................................................................  2  0
    ........................ugcugacguuugauccuugagc....................................................................................  1  0
    ..........................cugacguuugauccuugagc....................................................................................  1  0
    ..............................................agagguugccuuuggugaau................................................................  1  0
    ..............................................agagguugccuuuggugaauu...............................................................  2  0
    ..............................................agagguugccuuuggugaauuc..............................................................  5  0
    ..............................................agagguugccuuuggugaauucg.............................................................  3  0
```
